# Optical-Interferometry-Based CMOS-MEMS Sensor Transduced by Stress-Induced Nanomechanical Deflection

**DOI:** 10.3390/s18010138

**Published:** 2018-01-05

**Authors:** Satoshi Maruyama, Takeshi Hizawa, Kazuhiro Takahashi, Kazuaki Sawada

**Affiliations:** 1 AIST-TUT Advanced Sensor Collaborative Research Laboratory, Toyohashi University of Technology, Toyohashi, Aichi 441-8580, Japan; 2 Department of Electrical and Electronic Information Engineering, Toyohashi University of Technology, Toyohashi, Aichi 441-8580, Japan; takahashi@ee.tut.ac.jp (K.T.); sawada@ee.tut.ac.jp (K.S.); 3 Electronics Inspired-Interdisciplinary Research Institute (EIIRIS), Toyohashi University of Technology, Toyohashi, Aichi 441-8580, Japan; hizawa@ts.tut.ac.jp; 4 JST Precursory Research for Embryonic Science and Technology (PRESTO), Tokyo 102-0076, Japan

**Keywords:** Fabry–Perot interference, microelectromechanical systems (MEMS), complementary metal oxide semiconductor (CMOS)

## Abstract

We developed a Fabry–Perot interferometer sensor with a metal-oxide-semiconductor field-effect transistor (MOSFET) circuit for chemical sensing. The novel signal transducing technique was performed in three steps: mechanical deflection, transmittance change, and photocurrent change. A small readout photocurrent was processed by an integrated source follower circuit. The movable film of the sensor was a 350-nm-thick polychloro-para-xylylene membrane with a diameter of 100 µm and an air gap of 300 nm. The linearity of the integrated source follower circuit was obtained. We demonstrated a gas response using 80-ppm ethanol detected by small membrane deformation of 50 nm, which resulted in an output-voltage change with the proposed high-efficiency transduction.

## 1. Introduction

Microelectromechanical systems (MEMS)-based sensors allow the real-time detection of various parameters such as pressure, acceleration, force, and surface stress, which contributes to the Internet of Things (IoT) realm. As a sensor for measuring not only physical quantities but also biomolecules and chemical substances, MEMS sensors that capture the mechanical response to the adsorption of molecules are used to detect various molecules as well as the mechanical and electrical changes in structures due to adsorption, which can be observed in real time. In particular, a method for accurately and quickly inspecting biomolecules such as diseased proteins (biomarkers) from gas molecules contained in blood and exhaled gases has attracted attention as a simple diagnostic method for diseases.

Technologies using sensor elements miniaturized and arrayed by semiconductor microfabrication technology can detect gas molecules and biomolecules such as DNA [1,2], tumor markers [3,4], Escherichia coli [5], viruses [6,7], and gas molecules [8,9,10,11,12,13]. It is expected that the parallel detection and verification of different molecules can be performed promptly with such technologies [2,8,9]. A chemical and bio sensor using MEMS structures including a system for detecting a static deflection of the dynamic system and a micro-mechanical structure to detect a change in the mechanical resonance frequency has been proposed [14,15,16,17,18,19]. In a resonant chemical sensor, a functional film that specifically absorbs a molecule is formed on a silicon resonator, and the change in resonance frequency after the absorption of molecules is captured. Capacitive sensors [10,11] and piezoresistive sensors [12,13] have been reported for driving an MEMS resonator. In a capacitive sensor, an electrostatic gap is provided between the substrate and the sensing diaphragm, and a silicon thin film resonates and vibrates to successfully detect ppb-order substances such as chemical weapons [10] and carbon dioxide [11]. In a piezoresistive sensor, AIN and PZT are deposited on the movable film, the silicon bridge is subjected to resonant vibration, and frequency detection is performed by the vibration of the piezoelectric film. However, in such a vibration-type chemical sensor, an oscillation circuit is required for each sensor for frequency analysis, making the system complicated. On the other hand, surface sensors that detect static deformation due to the adsorption of molecules use the principle of detecting surface stress changes due to the repulsive force of adsorbed molecules. Compared to a resonance sensor, a surface stress sensor has less performance deterioration due to the influence of viscosity; therefore, this sensor can be used in various environments. Previously, the detection of alkanethiol adsorption using an atomic force microscope (AFM) cantilever has been reported [20,21]. A surface stress sensor that performs electrical detection with a chip has been also proposed for detecting a change in the film resistance with a piezoresistive element integrated in the movable film to detect a change in electrostatic capacitance. However, the conventional surface stress sensor responds to the displacement of the membrane in inverse proportion in the case of an electrostatic sensor and proportionally in the case of a piezoresistive sensor; therefore, it is difficult to improve the signal conversion efficiency. Further, in the case of an electrostatic sensor, the detection circuit is composed of an amplifier and an analog to digital converter as a discrete circuit, and it is connected to the sensor chip by a flexible, flat cable.

The authors have previously reported a method to fabricate an optical-interference-type surface stress sensor for improving the signal conversion efficiency of the surface stress sensor [22]. Compared to the conventional MEMS chemical sensor, our sensor improved the signal conversion efficiency by utilizing an exponential change of optical interference characteristics and showed analytical improvement by two orders of magnitude. In addition, since the MEMS light interference type sensor has a feature in the structure and the sensor section is a photodiode, this sensor can be integrated with a complementary metal oxide semiconductor (CMOS) image sensor. The Fabry–Perot optical interference surface stress sensor can expand to the voltage output of the sensor response with only the buffer amplifier, and it can be extended for the multiple detection and imaging of biomolecules to measure gas distribution by using CMOS image sensor technologies.

In this paper, we propose a method to convert the displacement of the movable film into a light intensity change by using optical interference, in which the flexible movable film on the photodiode has a hollow structure, and to convert the light-intensity change into an opto-electric signal with a photodiode. We demonstrate that the sensor using the proposed method can be applied as a gas molecule detection sensor. By using optical interference, we succeeded in obtaining a large output voltage for a slight deformation of several tens of nanometers. The Fabry–Perot interferometer sensor is expected to use sensor characteristics of one pixel for chemical imaging as a gas image sensor with high signal conversion efficiency. Compared with the previously reported sensor [22], the proposed sensor is integrated with a source follower circuit with high input impedance to make the sensor less susceptible to external influences.

## 2. Design

**Figure 1:**
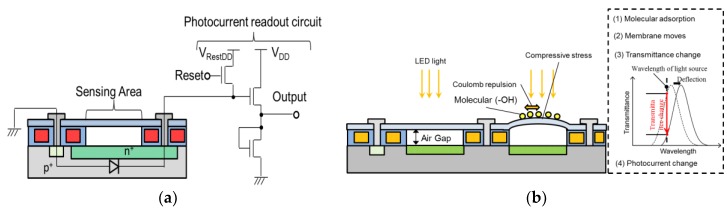
Circuit diagram of the Fabry–Perot sensor with the source follower circuit. (**a**) Schematic view of the microelectromechanical systems (MEMS) Fabry–Perot interferometric surface stress sensor and (**b**) deformation model of the movable film due to Coulomb repulsion.

**Figure 2:**
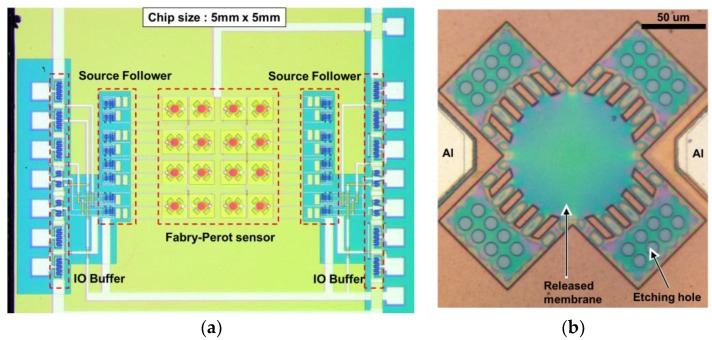
Photographs of the Fabry–Perot sensor interferometer. (**a**) The complementary metal oxide semiconductor (CMOS)-MEMS-based Fabry–Perot interferometer sensor with readout photocurrent circuits and (**b**) close-up view of the integrated MEMS Fabry–Perot sensor.

The Fabry–Perot optical interference surface stress sensor of this study is a hollow-structure MEMS that forms a photodiode and a source follower circuit using a 4-inch P-type (100) silicon wafer. Figure 1 shows a schematic illustration of the MEMS Fabry–Perot interferometric surface-stress sensor with a MOSFET signal processing circuit. A photodiode is fabricated in the silicon substrate as a detector. The movable film of this sensor consists of polychloro-para-xylylene (parylene-C), which has a Youngs’ modulus two orders lower than that of silicon, and the amount of deformation with respect to surface stress is expected to be greater with this film compared to that with conventional movable films. The parylene-C film is suspended over the silicon photodiode with an air gap. The Fabry–Perot interferometer consists of a thin flexible film, an air gap, and a silicon substrate with a silicon dioxide layer used for the passivation of the photodiode. The thickness of each layer determines the interferometric wavelength. When molecules are immobilized on the flexible film, the membrane deforms in the upward direction by the electrostatic repulsive force of adsorbed molecules, changing the Fabry–Perot interference wavelength. The mechanism of molecular adsorption at the movable film of the sensor will be explained in this paper. After the attachment treatment of an amino group (–NH_2_) on the movable film, the sensor is placed on a petri dish with alcohol and sealed. Since this movable membrane structure is restricted in its surroundings, when a hydroxyl group (–OH) contained in alcohol hydrogen bonds with an amino group, the repulsive force between the molecules is transmitted as compressive stress to the diaphragm and the central portion of the membrane swells. The interference wavelength changes, when irradiated with a monochromatic light source such as a light-emitting diode (LED), and the transmittance of the incident wavelength changes. This change in transmitted light intensity is read as a change in the photocurrent by the photodiode, and the presence or amount of a molecule can be quantitatively evaluated by a voltage signal passed through the signal processing circuit. Figure 2 shows the MEMS light-interference surface stress gas sensor of this study. The chip size is 5 mm^2^, with a sensor in the center part, a source follower circuit as the signal processing circuit on the right and left, and an electro static discharge (ESD) protection circuit. As the movable film of the sensor bulges, as shown in Figure 2b, the interference wavelength of the Fabry–Perot interferometer changes. The optical multilayer film of the Fabry–Perot interferometer consists of silicon-oxide film, air, and parylene.

**Figure 3:**
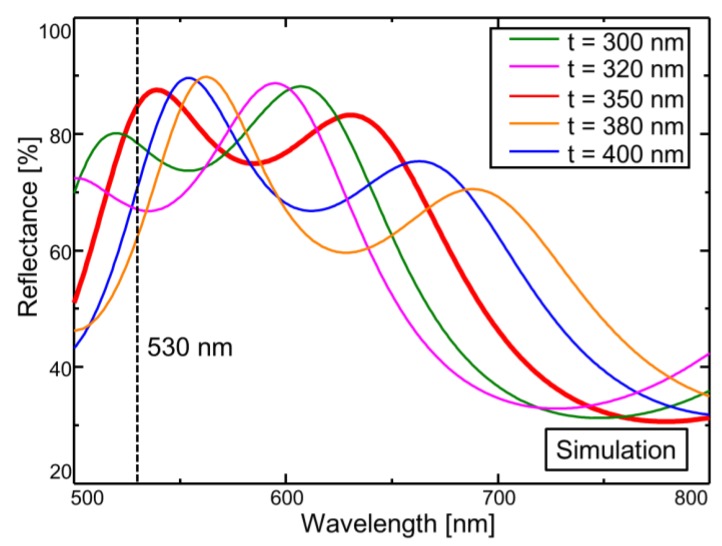
Reflectance spectrum of the Fabry–Perot interferometer for different thicknesses of the parylene-C film. The optical multilayer films are designed to use a 350-nm-thick parylene-C film and 400-nm-thick silicon-dioxide film with an air gap of 300 nm.

The optical multilayer films in this paper were designed to use 350-nm-thick parylene-C and 400-nm-thick silicon dioxide layers with an air gap of 300 nm. Figure 3 shows the difference in light reflection spectrum corresponding to the film thickness of parylene-C obtained using optical analysis software (RSOFT DiffractMOD). When the light frequency is 500 nm or less, the responsivity of the photodiode is low. Therefore, the parylene-C thickness was set to 350 nm so that the peak point of the spectrum was 500 nm or greater. The air gap was set to 300 nm in consideration of the selectivity ratio of sacrificial layer etching, under the same condition as the polysilicon of the gate. Considering the etching of the silicon-dioxide layer in all processes, the thickness was set to 400 nm. By using an LED with a wavelength of 530 nm, which is near the peak point, based on the analysis results, the photocurrent change associated with the spectral shift of the MEMS sensor can be readout. Therefore, the LED light source irradiating the sensor was set to 530 nm in this paper. The LED spectrum has a full width at half maximum (FWHM) of 40 nm, which is sufficiently small compared to that of the interference spectrum of the MEMS interferometer using a low-reflectance material. The typical FWHM of the parylene-C-based interferometer was found to be approximately 100 nm, as shown in Figure 3.

## 3. Fabrication

**Figure 4:**
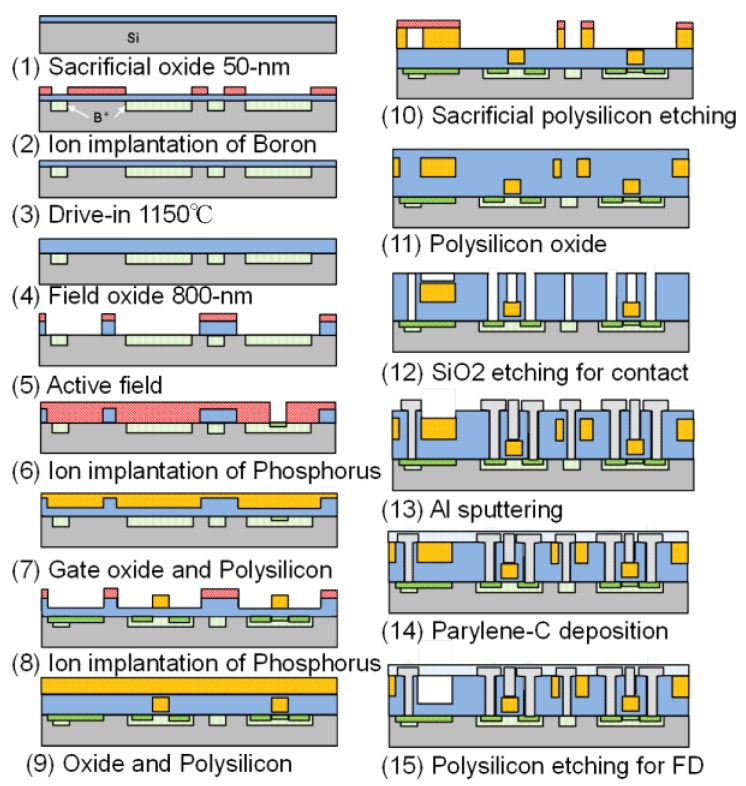
Fabrication process overview of the MEMS Fabry–Perot interferometer sensor with an integrated source follower circuit.

The fabrication process overviews of the Fabry–Perot optical interference surface stress sensor are shown in Figure 4. In this process, a 4-inch P-type silicon substrate was used. A sensor is fabricated as shown in the process flow. First, a dry oxide film was formed as a protective film for ion implantation. Next, threshold adjustment of the substrate was performed by ion implantation (B+), as well as lithography of the diffusion region. A polycrystalline silicon layer with a thickness of 5000 Å was deposited by low-pressure chemical vapor deposition (LP-CVD), and a gate electrode of the transistor was formed. Then phosphorus ions were implanted into the N layer of the photodiode, the source-drain region of the MOS transistor, and polysilicon. Polysilicon was deposited again and etched with xenon difluoride gas to create the hollow structure of the sensor. Subsequently, the surface of the sacrificial layer was oxidized. The air gap was set to 300 nm in consideration of the selectivity ratio of sacrificial layer etching, under the same condition as the polysilicon of the gate. A thermal oxide film was used as a passivation layer of the photodiode and an interlayer insulation film of the MOS because tetraethyl orthosilicate (TEOS) has a low etching selectivity for xenon difluoride; TEOS has been generally used for interlayer insulation for standard very large scale integration (VLSI) circuits. On the other hand, no influence of electrical properties of the MOS transistor insulated with thermal oxide was expected. In this paper, we propose an MOS transistor process that does not use TEOS, considering that the selection ratio of xenon difluoride gas decreases in TEOS during sacrificial layer etching. Thereafter, the oxide film on the sensor surface and the contact portion is removed, and Al–Si aluminum wiring is performed. After silane coupling treatment, parylene-C is deposited. Parylene-C is released after an etching hole is formed.

**Figure 5:**
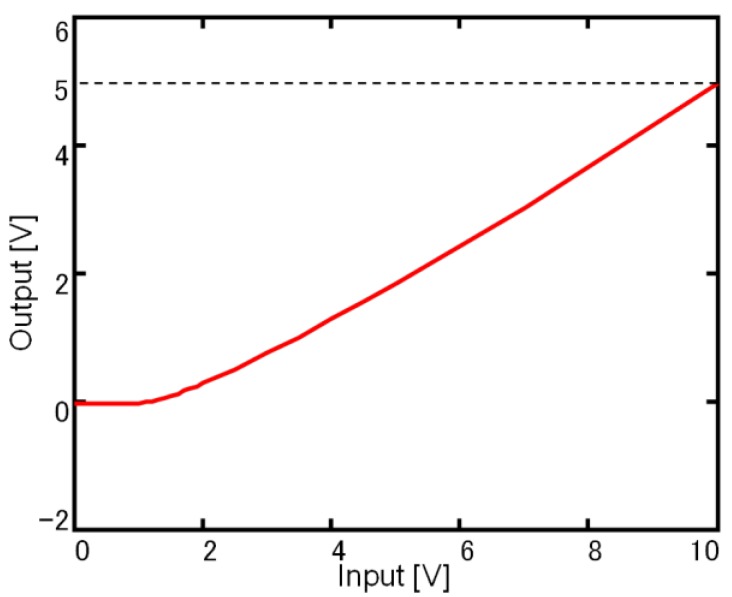
Fundamental characteristics of the negative-channel metal oxide semiconductor (NMOS) source follower circuit fabricated without using tetraethyl orthosilicate (TEOS).

Figure 5 shows the basic characteristics of a negative-channel metal oxide semiconductor (NMOS) source follower circuit fabricated by a process that does not use TEOS. The driving voltage is a DC voltage of 5 V, and the applied gate voltage is a linear voltage of 0 to 10 V. We confirmed the operation as a source follower circuit and demonstrated the CMOS-first-MEMS-last integration process by adapting the thermal-oxide passivation for the MOS transistors and the photodetector of the MEMS sensor.

## 4. Results and Discussion

**Figure 6:**
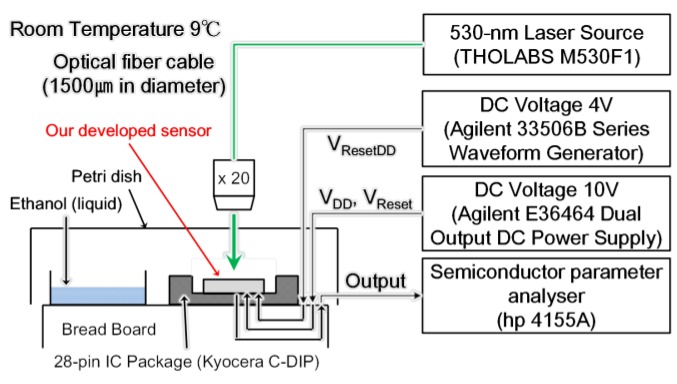
Schematic diagram of the experimental set up.

**Figure 7:**
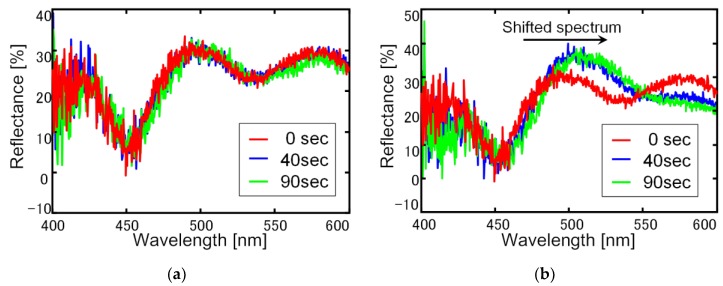
Change of the light reflection spectrum. (**a**) Sensor without parylene-AM and (**b**) sensor with parylene-AM.

**Figure 8:**
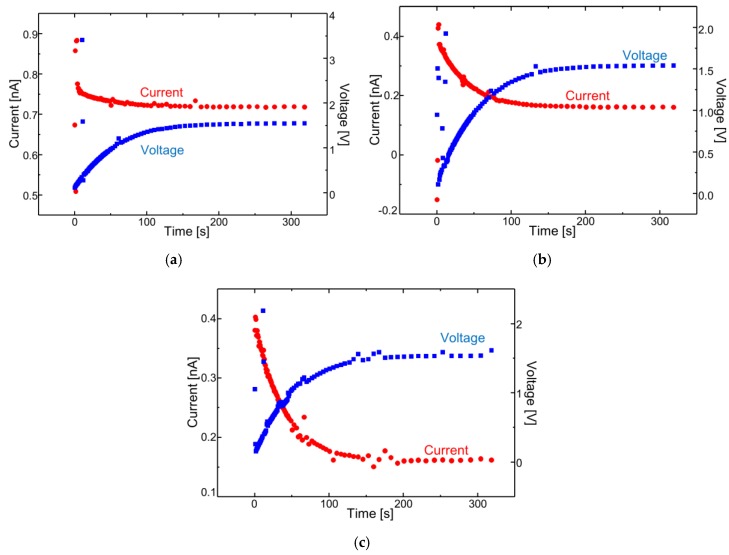
Time-measurement result of current and voltage in alcohol gas. (**a**) Ethanol, (**b**) methanol, and (**c**) isopropyl alcohol.

In this paper, a change of the light reflection spectrum of the sensor and the light signal received by the sensor section were reflected as a change in the current-voltage characteristics of the signal processing circuit, and the voltage-time change was measured using ethanol. The experiments were conducted at one concentration, and it was confirmed whether membrane deformation due to gas-molecule adsorption could be transduced. The reflectance of the sensing area was measured using a spectrometer (USB2000 Ocean Optics, Tokyo, Japan) with a white light source. The reflected light was collected by a microscopic objective lens and coupled into a fiber. The experimental set-up is shown in Figure 6. An LED light source (Thorlabs M530F1, Tokyo, Japan) with a wavelength of 530 nm and a power of 91 µW was used as the laser light source. First, a change in the light reflection spectrum was observed in a state where a sensor, before the vapor deposition of amino-methyl functionalized poly(para-xylylene) (diX-AM) on a movable film, was placed in an ethanol atmosphere for 90 s. It was confirmed that no change occurred in the movable film before the diX-AM film was deposited, as shown in Figure 7a. Figure 7b shows the change in the light reflection spectrum when a diX-AM film (thickness of approximately 10 nm to 20 nm) was evaporated on the same sensor and the chip was placed in an ethanol atmosphere for 90 s. Although the peak shifted slightly by approximately 50 nm, a sufficient change in current and voltage can be expected with this method. The results of voltage-time change measurement are shown in Figure 8. The change in the reflection spectrum of light over time was confirmed. When both current and voltage were measured as output signals, it was confirmed that the current decreased with time and the voltage increased. When the light reflection spectrum shifts from the structure of this sensor to the longer wavelength side, the output current decreases and the output voltage increases. The results of current measurement and voltage measurement are shown in order to confirm the voltage conversion of the output current of the photodiode by the integrated source follower circuit.

In addition, the output waveform has a relation of *x*-axis inversion between current and voltage. Since the sensor was initialized within approximately 30 s of release to the atmosphere, the gas response of methanol and IPA was acquired in addition to that of ethanol gas. As shown in Figure 8b,c, time-lapse measurements of current and voltage were also made using methanol and isopropyl alcohol in addition to ethanol. As a result of measuring the gas concentrations using a gas detector (RIDGID micro CD-100 Combustible Gas Detector), the concentrations of ethanol, methanol, and IPA were approximately 80 ppm, 900 ppm, and 800 ppm, respectively. Each concentration is the difference of volatilization volume of the solution with the same volume (3 mL).

**Figure 9:**
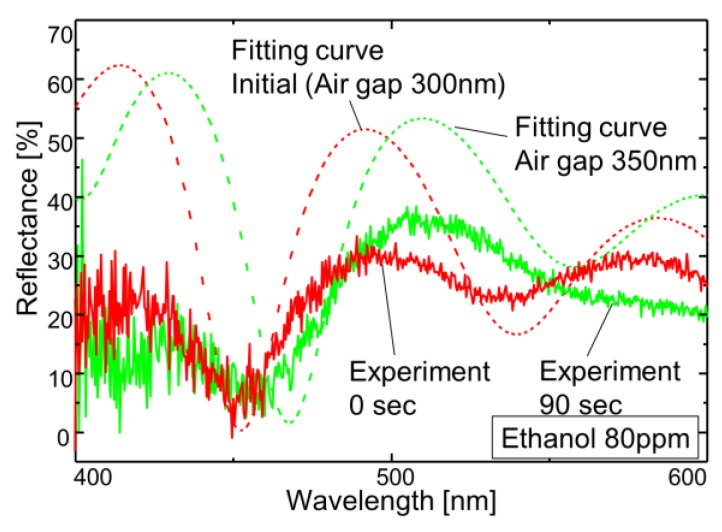
Reflectance spectrum of the Fabry–Perot interferometer with a change in the air gap. The optical multilayer films are designed to use 350-nm-thick parylene-C and 400-nm-thick silicon dioxide with air gaps of 300 nm and 350 nm.

**Figure 10:**
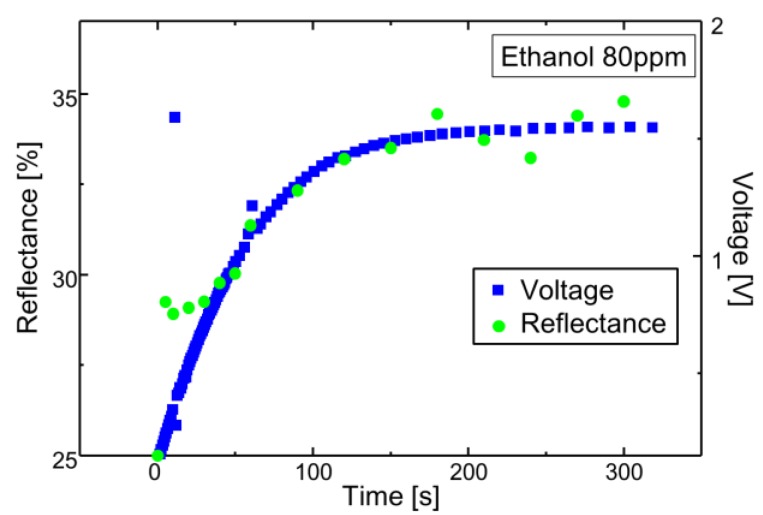
Comparison between reflectance and readout voltage.

Figure 9 shows the optical interference characteristics of the fabricated sensor using ethanol with a concentration of 80 ppm as well as fitting curves for different air gaps. The analysis parameters are the results obtained when the thickness of the parylene-C film was 350 nm, that of the silicon oxide film was 400 nm, and the air gap changed from 300 nm to 350 nm. The deviation of light intensity is assumed to be due to surface scattering because the short-wavelength light suffers from the surface roughness of the mirror. In this sensor, a change of approximately 1 V is obtained at a displacement of approximately 50 nm, and the conversion efficiency is good. It is considered that the hydroxyl group (–OH) of the alcohol and the amino group (–NH_2_) of the diX-AM membrane were hydrogen bonded and that the molecules adhered to the sensor film and repelled each other, whereby the membrane bulged. Figure 10 compares the light reflectance with the output voltage when the sensor is irradiated with a 530-nm laser light source. Reflectance at 530 nm was supposed to increase by the spectral shift associated with membrane deformation. The output voltage of the source follower circuit can be detected with respect to the change in reflectance. Hence, the slight deformation of the 50-nm membrane that adsorbs gas molecules was converted to a voltage of 1.5 V in the same manner as in a CMOS image sensor.

## 5. Conclusions

We have developed a CMOS-MEMS-based sensor that utilizes the optical-transmittance change via Fabry–Perot interference to enhance conversion efficiency. In this paper, we proposed a method to convert the displacement of a movable film into a light-intensity change by using optical interference, where the flexible movable film on the photodiode has a hollow structure, and to further convert the light-intensity change into an opto-electric signal with a photodiode. Consequently, the proposed Fabry–Perot interferometer sensor allows sensitive gas-molecule detection. The developed sensor can be integrated with a CMOS image sensor, and we experimentally showed the scalability to array sensors by simplifying the circuit as well as the feasibility of gas imaging.
